# A Method for Calculating Small Sizes of Volumes in Postsurgical Thyroid SPECT/CT Imaging

**DOI:** 10.3390/life15020200

**Published:** 2025-01-29

**Authors:** Elena Ttofi, Costas Kyriacou, Theodoros Leontiou, Yiannis Parpottas

**Affiliations:** 1 Department of Electrical Engineering, Computer Engineering & Informatics, Frederick University, Nicosia 1036, Cyprus; eng.kc@frederick.ac.cy; 2 Information Technology Department, German Medical Institute, Limassol 4065, Cyprus; 3 Frederick Research Center, Nicosia 1036, Cyprus; t.leontiou@frederick.ac.cy; 4 Department of Mechanical Engineering, Frederick University, Nicosia 1036, Cyprus

**Keywords:** thyroid–neck phantom, small thyroid remnants, volume calculation, postsurgical diagnostic thyroid imaging, nuclear medicine

## Abstract

Differentiated thyroid cancer treatment typically involves the surgical removal of the whole or largest part of the thyroid gland. Diagnostic procedures are useful both before and after treatment to determine the need for radioiodine ablation, re-stage the disease, monitor disease progression, or evaluate treatment efficacy. SPECT/CT imaging can be utilized to identify small, distant iodine-avid metastatic lesions and assess their uptake and volume for the above purposes as well as for performing lesion-based dosimetry when indicated. The objective of this study was to develop and validate a method for calculating small sizes of volumes in SPECT/CT imaging as well as to perform calculations utilizing I-131 and I-123 postsurgical SPECT/CT images from a neck–thyroid phantom. In this approach, the calculated volume was unaffected by radiation spillover from high-uptake voxels since it was the result from the successive application of the gray-level histogram technique to SPECT and CT 3D matrices. Beforehand, the SPECT 3D matrix was resized and aligned to the corresponding CT one. The method was validated following the clinical protocols for postsurgical thyroid imaging by using I-123 and I-131 scatter and attenuation-corrected SPECT/CT images from a neck–thyroid phantom. The phantom could accommodate two volumes of different sizes (0.5, 1, 1.5, 3, and 10 mL) and enclose anatomical tissue-equivalent main scattering structures. For the 0.5 and 10 mL volumes, the % differences between the actual and the calculated volumes were 15.2% and 1.2%, respectively. Radiation spillover was only present in SPECT images, and it was more profound at higher administered activities, in I-131 than in I-123 images, and in smaller volumes. When SPECT/low-dose-CT imaging is performed, this method is capable of accurately calculating small volumes without the need of additional modalities.

## 1. Introduction

Differentiated thyroid cancer (DTC) treatment typically involves the surgical removal of the whole or the largest part of the thyroid gland. Subsequent radioiodine therapy (RAIT) may be administered to selectively irradiate thyroid remnants, microscopic DTC, or non-resectable or incompletely resectable DTC [1]. Pre- or post-treatment diagnostic procedures are important to determine radioiodine ablation, re-stage the disease, and monitor disease progression or treatment efficacy [2,3,4,5].

Whole-body scintigraphy (WBS) is an essential diagnostic tool in the follow-up of DTC patients with high or intermediate risk (higher-risk features) of persistent disease [1]. It is particularly useful for detecting residual thyroid tissue, local recurrences, and distant metastases after thyroidectomy, both before and after RAI therapy. WBS is also recommended [6,7] for patients with positive or rising thyroglobulin (Tg) levels during follow-up and negative findings in cervical ultrasonography. Even a positive WBS or cervical ultrasonography result does not exclude the presence of non-iodine-avid tumors, which may require functional metabolic imaging using FDG PET/CT.

SPECT/CT imaging with I-123 or low-activity I-131 can focus on and evaluate regions of ambiguous findings from WBS. It can determine the extent of thyroid bed uptake after surgery and detect distant iodine-avid metastases, which can be factors in deciding whether RAIT should be administered and determining the appropriate I-131 dose [8]. Thyroid bed uptake may be particularly useful in cases in which the extent of the thyroid remnant cannot be accurately determined from the surgical report or cervical ultrasonography [1]. SPECT/CT imaging is capable of identifying and characterizing small lesions or lesions in anatomically complex regions due to its ability to precisely localize lesions and quantify lesion-specific iodine uptake. It can also detect pulmonary micrometastases that are too small to appear on routine chest X-rays or remain undetected on CT [2]. Additionally, it can identify bone metastases at an early stage, even before cortical disruption becomes visible on bone X-rays or CT [2].

DTC management guidelines [1] propose the selective use of radioiodine ablation for patients classified even as low risk or intermediate risk for DTC recurrence or mortality. Avram et al. [9] demonstrated the contribution of pre-ablation I-131 planar imaging combined with SPECT/CT in the staging of DTC patients after surgery. The identification of regional and distant metastases prior to RAI therapy significantly alters patient management. RAIT is most effective for smaller metastatic deposits; therefore, the early detection of regional and distant metastases is crucial for successful therapy [10,11].

In DTC, several factors influence the decision to perform ablation, including the number of small iodine-avid metastatic lesions, the type of thyroid cancer, lesion location and size, disease progression risk, lesion growth, structural or functional risks posed by metastases, and elevated thyroglobulin levels. The ATA guidelines [1] recommend considering intervention for metastatic lesions ≥1 cm to prevent progression, especially if iodine avidity is confirmed or there is a structural impact. Small iodine-avid lung metastases are often ablated with radioactive iodine, as they typically respond well [1], whereas small bone metastases are usually targeted due to their potential to cause structural damage over time.

Activity quantification within lesions and the volume (mass) of lesions are useful for optimizing DTC procedures. A study [12] aimed to establish a numerical relationship between serum Tg levels and tumor mass in diagnostic procedures using NaI-124 PET/CT and FDG PET/CT. Although a positive correlation was observed, the estimated tumor mass for a given Tg level exhibited a wide range. Consequently, this approach has limited value in clinical applications.

Pre-therapeutic dosimetry can determine the administered activity, while post-therapeutic dosimetry evaluates the energy dose absorbed by tissue. Complete dosimetry requires measurements of the target mass and the time-dependent iodine uptake within this mass. The activity needed to achieve a specified absorbed dose is linearly dependent on the target mass [13]. A position paper [14] suggests that individual dosimetry should be considered for high-risk DTC patients. It also emphasizes that dosimetry using diagnostic postoperative radioiodine imaging helps to avoid exceeding organ-specific dose limits, particularly in patients with renal impairment or diffuse lung metastases.

Lassmann et al. [15] note that empiric fixed activities do not attempt to determine either the minimal radioiodine activity required to deliver a lethal dose or the maximum reasonably safe absorbed dose. While the fixed activity-based approach for thyroid cancer treatment is simpler and often below toxicity limits, it does not account for patient individuality, which can result in over-treatment or, more commonly, under-treatment of individual patients [15]. Furthermore, administering multiple smaller doses may offer a lower therapeutic benefit compared to delivering the same total dose in a single administration [16].

Considering the above, an accurate calculation of lesion volume is desirable, particularly if it can be determined directly from SPECT/CT imaging when performed, without the need for additional modalities. A number of researchers developed methods for automatic or semi-automatic estimation of volumes and validated them by using physical phantoms and planar scintigraphy or SPECT images [17,18,19,20]. Automatic and semi-automatic segmentation techniques use active contour models on surfaces or volumes [21,22,23,24]. Automatic segmentation uses an active contour model with no or minimal input from user, relying on pre-defined parameters and initializations. Semi-automatic segmentation allows users to provide an initial contour or points to guide the model. Then, the contour evolves to minimize a predefined function which makes the contour to move toward the object boundaries.

Mortelmans et al. [17] developed a semi-automated method using the gray-level histogram (GLH) to apply thresholds for thyroid volume determination in Tc-99m SPECT images, and compared it to the fixed threshold approach. The latter approach employed a pre-defined fixed value where any voxel with a value below that threshold was considered as a surround or background, and any voxel with a value above that threshold was considered as object (volume). The adaptive threshold or GLH technique seeks to automatically determine a threshold that maximizes the separation between two classes identified in the image (e.g., object and surround) [25]. A physical phantom, consisting of cylindrical inserts of different sizes of volumes (9.8–202.5 mL) placed within a water tank, was employed for the evaluation of the method. The relative error was smaller when using the GLH technique than the fixed threshold approach, and it was reported to be 8.47%.

Zaidi [18] compared three methods (geometrical approximations, fixed threshold and GLH) to calculate volumes from Tc-99m SPECT images using phantoms, consisting of vials (16–75 mL) immersed within a solid Perspex water container. A relative error of 7.3% was achieved when using the GLH technique with scatter and attenuation correction.

Lyra et al. [19] developed a MATLAB algorithm to calculate different sizes of cylindrical phantoms (10–100 mL), immersed into a water tank, using Tc-99m SPECT imaging. They determined the borders of the phantoms from transaxial tomographic images using a specific threshold value for each case. A relative error of 8% was reported for the volume.

Pacilio et al. [20] developed a MATLAB algorithm, called recovering iterative thresholding method, which implemented threshold-volume and recovery-coefficient calibration curves. They determined the volume of test objects (20–110 mL) within the Jaszczak phantom (PMMA cylinder) from Tc-99m SPECT images, and they reported a percentage difference of 10%.

These previous studies calculated the volumes from Tc-99m SPECT images using simple phantoms such as vials within water containers. However, the limited SPECT spatial resolution did not allow them to estimate accurately volumes of less than 10 mL due to the partial volume effect (PVE) [26,27]. It is important to develop a method to accurately calculate volumes of less than 10 mL and evaluate it using a realistic scenario with clinical protocols for I-131 and I-123 postsurgical SPECT/CT thyroid imaging and using a dedicated anthropomorphic neck–thyroid phantom that encloses anatomically small sizes of volumes as well as tissue-equivalent human-shaped main scattering structures [28].

The objective of this study was to develop and validate a method for calculating small sizes of volumes as well as to perform calculations utilizing I-131 and I-123 SPECT/CT images using an anthropomorphic neck–thyroid phantom.

## 2. Materials and Methods

### 2.1. Method for Calculating Small Sizes of Volumes

#### 2.1.1. Concept

The volume was calculated from the number of voxels, resulting from the successive application of the GLH technique to SPECT and CT 3D matrices, times the elementary CT voxel size. Before applying the GLH technique, the SPECT slices were resized and then aligned to the corresponding CT ones to ensure that each SPECT voxel was registered and corresponded to a CT voxel.

First, the GLH was applied to SPECT voxels defined by a volume of interest (VOI). The threshold was derived from the uptake values, and it was used to separate the volumes (V_SPECT_) from the background [25]. Second, the GLH was applied to CT voxels, defined by the corresponding V_SPECT_. In this case, the Hounsfield Unit (HU) was used to derive the threshold and further separate the volumes from the background.

This approach effectively eliminates the radiation spillover effect [29,30], which is observed in SPECT imaging when radiation from high-uptake volumes spreads into neighboring background voxels. Spillover creates a misleading appearance of activity in the background, leading to the overestimation of the volume when relying solely on SPECT imaging. In contrast, the volume is not affected by the spillover effect when it is calculated from CT images.

However, while the calculated volume is free from the radiation spillover effect, it still remains affected, to a lower extent, by the PVE due to the CT matrix size (512 × 512 pixels with a pixel size of 1.1 × 1.1 mm) rather than due to the SPECT matrix (128 × 128 pixels with a pixel size of 4.4 × 4.4 mm).

#### 2.1.2. MATLAB Calculations

**Figure 1:**
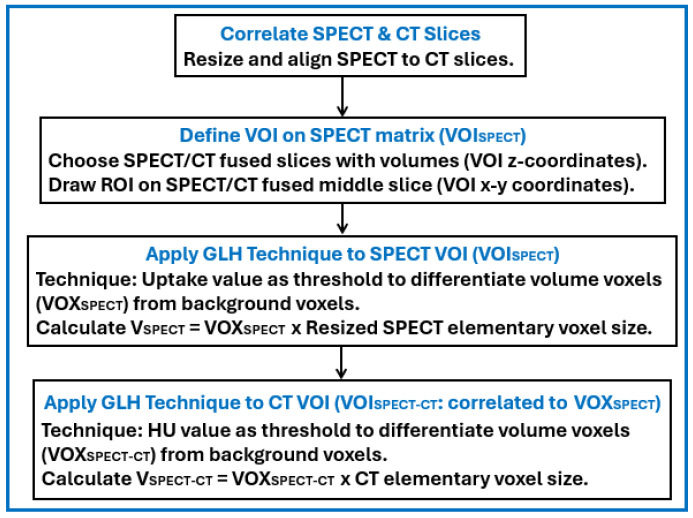
The main MATLAB tasks for the calculation of small volume sizes.

MATLAB (R2022a) [31] was utilized to calculate small volume sizes in SPECT/CT imaging. A number of key MATLAB tasks were performed such as correlating SPECT and CT matrices, defining the volume of interest, and applying the GLH technique twice in order to differentiate volumes from background. Figure 1 summarizes these tasks.

The first task was to correlate the SPECT and CT matrices. The MATLAB function ‘dicomread’ read the coordinates of the scanned area from the DICOM tags of the CT and SPECT slices. Next, the MATLAB function ‘imresize3’ resized the SPECT 128 × 128 matrices to the CT 512 × 512 matrices. Thus, an initial SPECT pixel with a size of 4.4 × 4.4 mm was resized to 16 pixels, each one with a size of 1.1 × 1.1 mm. Each of the 16 resized pixels contained the same uptake value as the initial pixel. After that, the SPECT and CT slices were aligned in the x, y, and z directions using the MATLAB function ‘circshift’. The output was a SPECT 3D matrix, with an elementary voxel size of 1.1 × 1.1 × 4.4 mm, aligned and correlated to the CT one.

The second task was to define a VOI on the SPECT matrix (VOI_SPECT_) for the first application of the GLH technique. For this purpose, the initial and last SPECT/CT fused slices, on which the volumes were observed, were chosen. Then, their middle slice was retrieved and a ROI was drawn around the high-uptake region. The VOI_SPECT_ was created by the x–y coordinates (ROI) and the z coordinates (initial and last slices).

**Figure 2:**
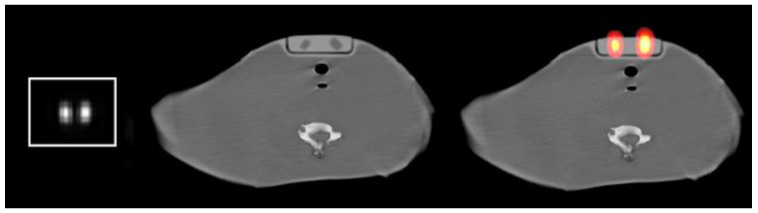
(**Left**) A scatter and attenuation-corrected I-131 SPECT middle slice from the neck–thyroid phantom with the 1.5 (left part on the image) and 3 mL (right part on the image) volumes, administered with 0.740 MBq and 1.295 MBq, respectively; (**middle**) the corresponding CT middle slice; and (**right**) the corresponding SPECT/CT fused middle slice. The middle slice is defined by the first and last slices on which the volumes are observed.

**Figure 3:**
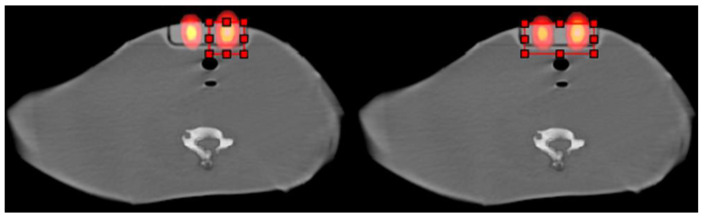
ROIs (red outline with dots) around (**left**) one and (**right**) both volumes on the SPECT/CT fused middle slice.

Figure 2 presents a scatter and attenuation-corrected I-131 SPECT middle slice from the neck–thyroid phantom with the 1.5 and 3 mL volumes and the corresponding CT and SPECT/CT fused middle slices. Figure 3 displays the ROIs around one and both volumes on the SPECT/CT fused middle slice.

The third task was to apply the GLH to VOI_SPECT_. The MATLAB function ‘multithresh’ [24], based on the Otsu theory [25], was utilized. The uptake values were used by the MATLAB function to derive the threshold for differentiating the volumes from the background. Any voxel with a value below the threshold was considered as background while any voxel with a value above the threshold was considered as volume. A distinct uptake difference was observed between the volumes and the background, as in diagnostic SPECT thyroid imaging, where the radiation in the background is usually 5–10% of the corresponding one in the volumes [28,32]. V_SPECT_ was calculated from the number of voxels, considered as volume, times the SPECT resized elementary voxel size.

**Figure 4:**
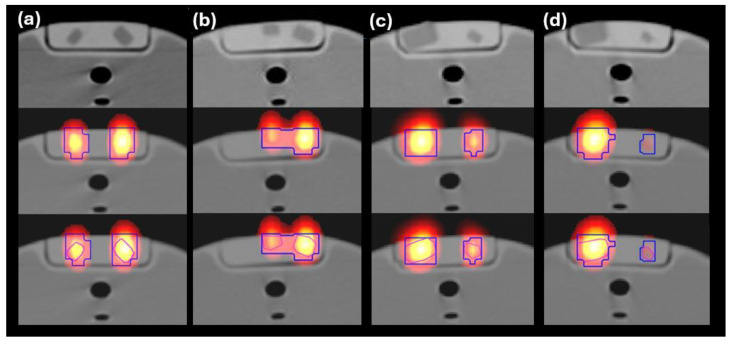
(**Top**) The CT middle slice from the phantom including two volumes. (**middle**, **bottom**) The corresponding I-131 SPECT/CT fused middle slice on which the V_SPECT_ (blue-colored outer loop) and V_SPECT-CT_ (purple-colored inner loop) are projected. V_SPECT_ and V_SPECT-CT_ are the calculated volumes from the SPECT and CT VOIs, respectively. From left to right of each image, the volumes are: (**a**) 1.5 and 3 mL with an 1 cm separation distance, (**b**) 1.5 and 3 mL with an 0.5 cm separation distance, (**c**) 10 and 1 mL, and (**d**) 10 and 0.5 mL.

**Figure 5:**
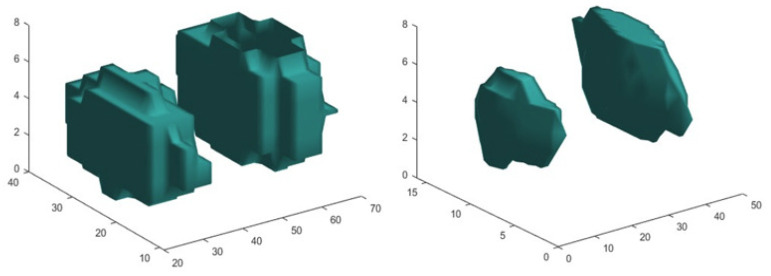
The 1.5 (left part on the image) and 3 mL (right part on the image) volumes from (**left**) V_SPECT_ and (**right**) V_SPECT-CT_.

The last task was to apply the GLH to a CT VOI (VOI_SPECT-CT_) using the same MATLAB function as above. The V_SPECT_ voxels were used to define VOI_SPECT-CT_, as SPECT and CT voxels were correlated beforehand. The HU was utilized to derive the threshold for differentiating the volumes from the background. In VOI_SPECT-CT_, unlike in VOI_SPECT_, the HU difference between the volumes and the background was not very well distinct. Hence, an adaptive HU threshold was utilized that was determined by reading the HU distribution within the VOI_SPECT-CT_, identifying similar HU regions, and calculating the minimum and maximum percentiles for each similar HU region. V_SPECT-CT_ was calculated from the number of voxels, considered as volume, times the CT elementary voxel size. V_SPECT_ and V_SPECT-CT_ are projected on the SPECT/CT fused middle slice in Figure 4, and they can be 3D-visualized in Figure 5.

A standalone fast software was developed to apply the above MATLAB calculations where the user can zoom in/out and navigate through the SPECT, CT, and SPECT/CT fused slices of Figure 2, draw a ROI on the slice of Figure 3, and zoom in/out and rotate the volumes of Figure 5. The number of detected volumes, their V_SPECT_ and V_SPECT-CT_ values, as well as their corresponding number of voxels can be archived by filling in customized fields and then saved.

#### 2.1.3. Dependencies of the Gray-Level Histogram Technique

The GLH technique depends on (a) the ROI size, (b) the derived threshold (uptake or HU) that separates the volume from the background, and (c) the separation distance between nearby volumes. The actual volume was always included within the ROI, as it was manually drawn on the SPECT/CT fused middle slice to enclose pixels with radiation, spillover radiation, and the surrounding background region. All acquired SPECT/CT images included two volumes.

The volumes were calculated using an ROI that included either one or both volumes, as shown in Figure 3. This is stated in the corresponding text related to each table.

For the same thresholding approach and dataset (matrix), the threshold value remains unchanged. Slight variations in the manually drawn ROI size (whether including one or two volumes) result in low standard deviation (SD) values for the calculated volume. Different administered activities for the same volume (e.g., 1.5 and 3 mL) may result in different calculated volumes because the datasets differ. For these cases, the SD values for the calculated volumes are reported or derived using error propagation.

The separation distance between the nearby volumes, reported in the Tables, ranged from 1 to 2 cm. Two more I-131 SPECT/CT acquisitions were performed with a separation distance of 0.5 cm between the nearby 1.5 and 3 mL volumes. The calculated volumes from these acquisitions were compared to the corresponding volumes with a separation distance of 1 cm between the nearby volumes (Figure 4a,b).

### 2.2. Validation Procedures

I-123 and I-131 SPECT/CT images from a neck–thyroid phantom with small sizes of volumes (0.5, 1, 1.5, 3, and 10 mL) were utilized to validate the method. The phantom was specifically designed for postsurgical thyroid imaging and was developed using 3D printing and molding techniques [28]. The phantom can accommodate two small sizes of volumes and can simulate different background-to-volume activity ratios. Other structures, such as the trachea, esophagus, cervical spine, and clavicle, were also anatomically enclosed within the phantom. Figure 2 presents slices from the phantom: a SPECT slice with the 1.5 and 3 mL volumes and the corresponding axial CT and SPECT/CT fused slices.

The dual-head GE Infinia Hawkeye 4 SPECT/4-slice CT hybrid scanner (GE Health care, Milwaukee, WI, USA) of the Bank of Cyprus Oncology Center was utilized to acquire the I-131 and I-123 images of the phantom. All acquisitions were performed following the clinical protocols. For I-131, two high-energy general-purpose (HEGP) collimators in 180° (H mode) orientation were used. SPECT data were acquired in 60 projections, 35 s per projection, over 180° of rotation thus covering an angular range of 360°, using a matrix size of 128 × 128. The data were reconstructed using the ordered-subset expectation maximization (OSEM) algorithm with two iterations and ten subsets. A Butterworth filter (cut-off: 0.48, power: 10) was applied on the reconstructed images. SPECT data were scattered corrected using the triple-energy window (TEW) method, as described by Hadjiconstanti et al. [33]. For the TEW method, three energy windows were set during the I-131 acquisitions: (a) a 10% main window around the 364 keV photopeak of I-131, (b) a lower-energy scatter window at 304.4–309.4 keV, and (c) a higher-energy scatter window at 418.6–423.6 keV.

I-123 acquisitions were performed using two low-energy high-resolution (LEHR) collimators. For the TEW scatter correction, the following energy windows were set: (a) a 10% main window centered over the 159 keV photopeak of I-123, (b) a lower-energy scatter window at 134.5–139.5 keV, and (c) a higher-energy scatter window at 177.1–182.5 keV [34]. The rest of the acquisition and reconstruction parameters were similar to the corresponding I-131 acquisitions.

CT images with 5 mm slice thickness were acquired using a matrix size of 512 × 512 and they were used for the attenuation correction of the SPECT images.

The activities of the I-123 and I-131 solutions were measured using a calibrated dose calibrator (Capintec CRC-15R, Capintec, Florham Park, NJ, USA). The activity concentration (MBq/mL) of the solution was determined by dividing the measured activity with the volume of the solution. The solution was then thoroughly mixed with water before being injected into the empty cavities (volumes and background) of the phantom. Subsequently, the filled cavities were thoroughly shaken to ensure a homogeneous activity distribution and to eliminate potential hot or cold spots. The administered activities within the phantom cavities are reported for the time of acquisition. The administered activities were selected to ensure a linear response of the SPECT/CT modality (count vs. activity) and correspond to activities used in diagnostic protocols [28,32,33].

V_SPECT_ and V_SPECT-CT_ were calculated, when a VOI included only one or two volumes, for different I-123 and I-131 administered activities and for different background-to-volume activity ratios (%Bkg = 0, 5, 10). The % differences between the actual volume (V) and V_SPECT-CT_ were reported. The volume ratio (R = V_SPECT_/V_SPECT-CT_) for the different sizes of volumes administered with I-123 and I-131 activities were also reported and compared. When a specific volume size appeared in multiple acquisitions with different administered activities, the average V_SPECT_ and V_SPECT-CT_ values along with their corresponding standard deviation (SD) were reported. For a specific volume size appearing in only one or two acquisitions, their average V_SPECT_, V_SPECT-CT_ values were obtained from five calculations of the same acquisition.

## 3. Results

Table 1 presents the number of I-123 and I-131 SPECT/CT acquisitions for different administered activities within a specific volume size and the % difference (%ΔV) between V and V_SPECT-CT_. V_SPECT-CT_ represents the average value when the VOI included only one volume, as explained in a previous section. %ΔV is larger for smaller volumes due to PVE [26,27]. Even though V_SPECT-CT_ was calculated by applying the GLH technique to a CT VOI with an elementary voxel size of 1.1 × 1.1 × 4.4 mm rather than to a SPECT VOI with an elementary voxel size of 4.4 × 4.4 × 4.4 mm, it is still affected, to a lesser extent, by PVE. Note that the 0.5 mL volume could be measured in three consecutive slices, and the 1 and 1.5 mL volumes in four and five consecutive slices, respectively.

Table 2 presents the calculated volumes V1_SPECT-CT_ and V2_SPECT-CT_ when the VOI included only one and two volumes, respectively, for the different sizes of volume observed on the I-123 and I-131 SPECT/CT images. Note that the VOI size, when including two volumes, was at least twice the size of the VOI when including only one volume. V1_SPECT-CT_ and V2_SPECT-CT_ represent the average values, as explained in a previous section. The corresponding % differences between V1_SPECT-CT_ and V2_SPECT-CT_ (%ΔV_1–2_) are also reported.

In all cases, the V1_SPECT-CT_ values are smaller than the V2_SPECT-CT_ values and closer to the V values. The reported %ΔV_1–2_ values are low (1.1–9.1%). The highest %ΔV_1–2_ value corresponds to the 0.5 mL volume administered with I-131, while the actual volume difference is 0.05 mL. The lowest %ΔV_1–2_ value corresponds to the 10 mL volume administered with I-123, while the actual volume difference is 0.11 mL. No significant difference was observed between the corresponding I-123 and I-131 %ΔV_1–2_ values.

From the V1_SPECT-CT_, V2_SPECT-CT_ and %ΔV_1–2_ values, it can be observed that the GLH technique is slightly more efficient when smaller VOIs are used and when larger volumes are calculated. However, applying the GLH technique simultaneously to both volumes is preferable, as it delivers acceptable results.

Table 3 presents the volume ratios (R = V_SPECT_/V_SPECT-CT_) calculated for the different sizes of volume observed on the I-123 and I-131 SPECT/CT images. R represents the average value when the VOI included only one volume, as explained in a previous section. In all cases, the R values are higher than the unity, since V_SPECT_ values are always higher than the corresponding V_SPECT-CT_ ones, mostly because of the radiation spillover effect presented in V_SPECT_ [29,30]. Both V_SPECT_ and V_SPECT-CT_ for smaller volumes were affected by the PVE but to a different extent, as previously explained (V_SPECT_ more than V_SPECT-CT_). The spillover effect in V_SPECT_ is more profound (a) at higher administered activities of the same radiopharmaceutical within the same volume size, (b) in I-131 than I-123 SPECT images of similar administered activities within the same volume size, and (c) in smaller volume sizes administered with similar activities and the same radiopharmaceutical.

**Figure 6:**
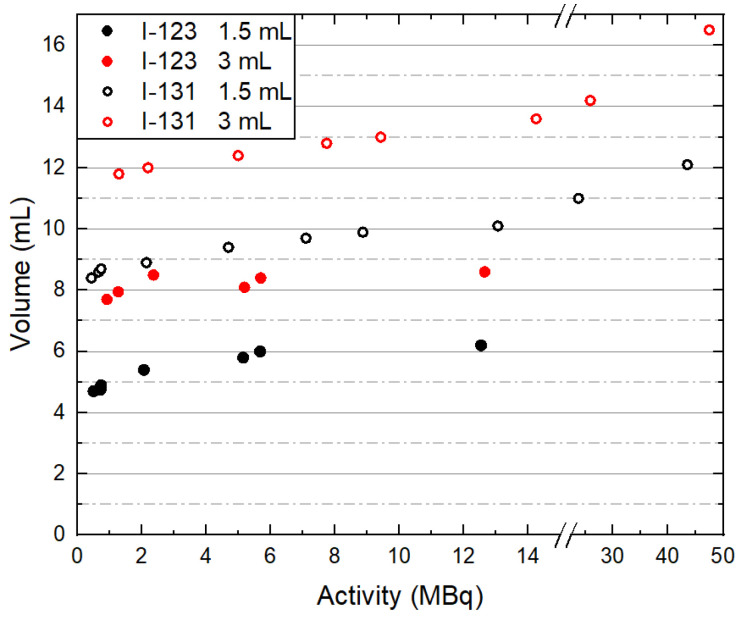
V_SPECT_ values with respect to the I-123 and I-131 administered activities within the 1.5 and 3 mL volumes.

For the 10 mL volume, where the I-123 and I-131 administered activities were comparable, the R(I-131) value is higher than the R(I-123) one. For the 1.5 and 3 mL volumes, the average R(I-131) values are higher than the corresponding average R(I-123) values because the I-131 images are more affected than the I-123 images by the spillover effect for similar administered activities, and also because higher I-131 administered activities were utilized for the SPECT/CT acquisitions (Figure 6). For the 0.5 and 1 mL volumes, the corresponding R(I-131) and R(I-123) values cannot be compared because higher I-123 than I-131 administered activities were utilized (Table 1).

Figure 6 presents the calculated V_SPECT_ values with respect to I-123 and I-131 administered activities within the 1.5 and 3 mL volumes. V_SPECT_ values are higher for higher administered activities within the same volume size and radiopharmaceutical due to the spillover effect. In addition, V_SPECT_(I-131) > V_SPECT_(I-123) for all administered activities, as previously explained.

Table 4 presents the calculated V_SPECT_ and V_SPECT-CT_ values for the 1.5 and 3 mL volumes observed on the I-123 and I-131 SPECT/CT images. The administered activity within the volumes was 0.37 MBq/mL, while the %Bkg was 0, 5, and 10. V_SPECT_ and V_SPECT-CT_ represent the values when the VOI included only one volume. The mean and SD values are also reported for the different volume sizes and different radiopharmaceuticals.

The % V_SPECT-CT_ difference among the different %Bkg, for the same volume size and same radiopharmaceutical, varied slightly in the range of 1–2.5%. However, the % V_SPECT_(I-123) differences between %Bkg of 0 and 10, for the 1.5 and 3 mL volumes, were 14.9% (0.7 mL actual difference) and 10.4% (0.8 mL actual difference), respectively. The corresponding % V_SPECT_(I-131) differences for the 1.5 and 3 mL volumes were 19.6% (1.8 mL actual difference) and 14.5% (1.9 mL actual difference), respectively.

V_SPECT_ values are increased as the background activity is increased. As expected, this increase is more profound in I-131 than I-123 SPECT images and corresponds to an actual difference of about 1 mL. This is due to the higher I-131 spillover out of the volume region but also in the volume region from the background radiation. In contrast, V_SPECT-CT_ is not influenced by the %Bkg since it does not depend on SPECT uptake values. This is also illustrated by the corresponding low SD values.

## 4. Discussion

When SPECT/CT imaging is utilized to identify small distant iodine-avid metastatic lesions, representing early-stage residual or recurrent disease, it is important to assess lesion uptake and volume for (a) monitoring disease progression or treatment efficacy, complementing serial serum Tg measurements, and (b) performing lesion-based dosimetry, when indicated, to determine the RAIT activities and/or evaluate the energy dose absorbed by tissue. This method can accurately determine small (0.5–10 mL or 1–2.7 cm in diameter) and larger lesion volumes using I-123 and I-131 SPECT/low-dose CT thyroid imaging without the need for additional modalities.

Previous studies [17,18,19,20] developed methods using the GLH technique for calculating volumes using Tc-99m SPECT imaging. An 8% relative error was reported for the smaller examined volume of 10 mL [19]. In this study, the validation was performed under a more realistic scenario compared to previous studies, in terms of the phantom, acquisitions, and radiopharmaceuticals used. For the smallest and largest examined volumes of 0.5 and 10 mL, the percentage differences between the actual and calculated volumes were 15.2% and 1.2%, respectively.

We were able to calculate much smaller volumes with greater accuracy than in previous studies because, unlike earlier methods that applied the GLH technique to SPECT images, the calculated volumes were (a) unaffected by the radiation spillover effect and (b) less affected by PVE. This improvement was achieved because the calculated volume was derived from the application of the GLH technique to a CT VOI with an elementary voxel size of 16 times smaller than the corresponding SPECT voxel size. Note that the GLH was initially applied to a resized SPECT VOI to define the corresponding CT VOI.

Radiation spillover in V_SPECT_ is more profound (a) at higher administered activities of the same radiopharmaceutical within the same volume size, (b) in I-131 than I-123 SPECT images of similar administered activities within the same volume size, and (c) in smaller sizes of volume administered with similar activities and the same radiopharmaceutical. I-123 compared to I-131 images present less radiation spillover because I-123 emits lower energy gammas, has better spatial resolution, and produces less scattering, all of which contribute to sharper images and a more accurate localization of radioactivity [28,35,36,37].

Even though higher background-to-volume activity ratios increase V_SPECT_, especially in I-131 compared to I-123 SPECT imaging due to the higher I-131 spillover in/out the volume region, V_SPECT-CT_ is not influenced by the background radiation since it is calculated from a CT VOI.

The SPECT/CT fused middle slice was utilized to either (a) draw a ROI around a volume, proceed with the calculation of the volume, and then repeat the same process for the other observed volumes, or (b) draw a ROI around all observed volumes and proceed with the simultaneous calculation of each volume. In this study, two volumes were presented in each SPECT/CT image. The volumes were calculated either by drawing a ROI that included only one or both volumes. Even though the size of the smaller ROI was less than half of the size of the larger ROI, the highest reported % difference between the corresponding calculated volumes, when drawing a small or large ROI, was 9% and it was reported for the 0.5 mL volume administered with I-131. The calculated volumes obtained by using a ROI containing a single volume was slightly more accurate than the corresponding volumes calculated by using a ROI containing two volumes. However, the latter approach is faster, and it still delivers good results.

As demonstrated, the GLH technique effectively separates two regions—volumes from background—even when the differences in uptake or HU are high or low. Specifically, the GLH was applied to SPECT images acquired over a wide range of administered activities within the volumes and using two radiopharmaceuticals (0.44–47.47 MBq of I-131 and 0.55–12.65 MBq of I-123), which resulted in varying degrees of radiation spillover. Additionally, the GLH was applied to CT images to distinguish two regions with low HU differences: 1050–1090 HU in volume region and 1115–1130 HU in the surrounding background (1000 HU in water). In this case, the GLH utilized an adaptive threshold determined by the minimum and maximum percentiles of each region, ensuring an accurate separation.

Even though the GLH technique can calculate multiple thresholds for images with more than two intensity classes, it would be preferable, when possible, to draw a ROI within the same anatomical structure, that is, to enclose only the volume(s) and the surrounding environment (two HU classes with even low HU difference). In most of the cases, the separation distance between two nearby volumes ranged from 1 to 2 cm. For a smaller separation distance (e.g., 0.5 cm, as shown in Figure 4b), while two volumes could be distinguished on a SPECT/CT fused slice, the algorithm could only process calculations when the ROI included both volumes. At a separation distance of 0.5 cm (Figure 4b) compared to 1 cm (Figure 4a), the calculated volumes (1.5 and 3 mL) were 10% less accurate. Hence, a separation distance between nearby volumes of at least 1 cm is considered as a limitation for accurate calculations.

The results can be reproduced on different SPECT/CT scanners and clinical settings for the same matrix sizes (SPECT: 128 × 128, CT: 512 × 512), CT slice thickness (5 mm), and elementary CT voxel size (1.1 × 1.1 × 4.4 mm). However, for a SPECT matrix size of 256 × 256, the CT slice thickness is recommended to be 2.5 mm.

This method complements broader research efforts in developing low-dose approaches for thyroid cancer management, including the use of iodine isoforms for theranostic applications and the potential utilization of nanoparticles as drug delivery vehicles, as the latter advancements enable the targeted transport of low doses of therapeutic agents directly to cancerous or inflamed tissues [38,39]. Furthermore, the results of this study, along with subsequent results by applying this method to other nuclear medicine imaging procedures, could contribute to ongoing investigations [40] into morpho-functional imaging with radiomics (leveraging AI algorithms) for enhancing targeted treatment management.

## 5. Conclusions

A method was developed and applied to accurately calculate SPECT/CT image volumes as small as 0.5 mL. It was validated using I-123 and I-131 postsurgical SPECT/CT thyroid imaging and a neck–thyroid phantom with small volumes as well as other tissue-equivalent main scattering structures.

It calculates volumes of 0.5 and 10 mL (1.0–2.7 in diameter size) with % differences of 15.2% and 1.2%, respectively. The calculated volume is not unaffected by the radiation spillover effect, while the extent of partial volume effect is reduced. Moreover, the calculated volume is not affected by the background radiation.

The GLH technique effectively distinguishes regions of multiple volumes from the background, even two regions with low HU differences. However, it is recommended to define regions of interest that enclose volumes within the same anatomical structure to ensure optimal accuracy.

When SPECT/low-dose-CT imaging is performed for the DTC management, this method can accurately calculate small lesion volumes without the need for additional modalities.

## Figures and Tables

**Table 1:** The number (No.) of I-123 and I-131 SPECT/CT acquisitions for different administered activities (A) within a specific volume size as well as the % difference (%ΔV) between the actual volume (V) and the average calculated volume (V_SPECT-CT_).

V (mL)	I-123			I-131		
	A (MBq)	No.	%ΔV	A (MBq)	No.	%ΔV
0.5	2.173	1	15.2	0.555	1	15.8
1	2.561	1	11.7	1.109	1	12.1
1.5	0.555–12.542	7	8.0	0.444–43.512	10	8.6
3	1.110–12.650	6	3.7	1.110–47.471	8	4.1
10	3.228, 3.581	2	1.2	3.700, 3.990	2	1.4

**Table 2:** The average calculated volumes V1_SPECT-CT_ and V2_SPECT-CT_, along with their SD, when the VOI included only one and two volumes, respectively, for the different volume sizes (V) observed on I-123 and I-131 SPECT/CT images as well as the corresponding % difference (%ΔV_1–2_) between V1_SPECT-CT_ and V2_SPECT-CT_.

V (mL)	I-123			I-131		
	V1_SPECT-CT_	V2_SPECT-CT_	%ΔV_1–2_	V1_SPECT-CT_	V2_SPECT-CT_	%ΔV_1–2_
0.5	0.576 ^1^	0.626 ^1^	8.8	0.579 ^1^	0.631 ^1^	9.1
1	1.117 ^1^	1.196 ^1^	7.1	1.121 ^1^	1.209 ^1^	7.9
1.5	1.621 ± 0.033	1.655 ± 0.036	2.1	1.630 ± 0.039	1.669 ± 0.045	2.4
3	3.113 ± 0.031	3.169 ± 0.035	1.8	3.123 ± 0.040	3.182 ± 0.046	1.9
10	10.120 ± 0.004	10.231 ± 0.006	1.1	10.140 ± 0.011	10.251 ± 0.017	1.1

^1^ SD < 0.001 (see Section 2.1.3).

**Table 3:** The average volume ratios (R = V_SPECT_/V_SPECT-CT_), along with their SD, calculated for the different volume sizes observed on the I-123 and I-131 SPECT/CT images.

V (mL)	R(I-123)	R(I-131)
0.5	12.04 ± 0.01 ^1^	9.84 ± 0.01 ^1^
1.0	7.89 ± 0.01 ^1^	8.71 ± 0.01 ^1^
1.5	3.32 ± 0.38	5.94 ± 0.72
3.0	2.63 ± 0.11	4.25 ± 0.49
10.0	1.50 ± 0.01	2.41 ± 0.07

^1^ SD values are low due to the low SD of V_SPECT_ and V_SPECT-CT_ (see Section 2.1.3).

**Table 4:** The calculated V_SPECT_ and V_SPECT-CT_ values for the 1.5 and 3 mL volumes, observed on the I-123 and I-131 SPECT/CT images, with an administered activity of 0.37 MBq/mL and different background-to-volume activity ratios (%Bkg) as well as the mean and SD values for the different volume sizes and radiopharmaceuticals.

V (mL)	%Bkg	I-123		I-131	
		V_SPECT_	V_SPECT-CT_	V_SPECT_	V_SPECT-CT_
1.5	0	4.711	1.614	8.601	1.623
1.5	5	5.225	1.630	9.841	1.642
1.5	10	5.413	1.626	10.292	1.664
Mean ± SD		5.116 ± 0.363	1.623 ± 0.008	9.578 ± 0.875	1.643 ± 0.020
3.0	0	7.703	3.094	11.602	3.116
3.0	5	8.221	3.098	12.981	3.141
3.0	10	8.508	3.165	13.521	3.152
Mean ± SD		8.144 ± 0.407	3.119 ± 0.039	12.701 ± 0.989	3.136 ± 0.018

## Data Availability

The data presented in this study are available upon request from the corresponding authors.

## Abbreviations

The following abbreviations are used in this manuscript:

%Bkg	Background-to-Volume Activity Ratio
%ΔV	Percentage Difference between V and V1_SPECT-CT_
%ΔV_1–2_	Percentage Difference between V1_SPECT-CT_ and V2_SPECT-CT_
3D	Three-Dimensional
CT	Computed Tomography
DICOM	Digital Imaging and Communications in Medicine
DTC	Differentiated Thyroid Cancer
FDG	Fluorodeoxyglucose
GLH	Gray-Level Histogram
HEGP	High-Energy General-Purpose
HU	Hounsfield Unit
I-123	Iodine 123
I-131	Iodine 131
LEHR	Low-Energy High-Resolution
MATLAB	Matrix Laboratory
NaI-124	Potassium Iodine 124
OSEM	Ordered-Subset Expectation Maximization
PET	Positron Emission Tomography
PMMA	Polymethyl Methacrylate
PVE	Partial Volume Effect
R	Volume Ratio: V_SPECT_/V_SPECT-CT_
RAIT	Radioiodine Therapy
ROI	Region-of-Interest
SD	Standard Deviation
SPECT	Single-Photon Emission Computed Tomography
Tc-99m	Technetium 99m
TEW	Triple-Energy Window
Tg	Thyroglobulin
V	Actual Volume
V1_SPECT-CT_	V_SPECT-CT_ When ROI Included One Volume
V2_SPECT-CT_	V_SPECT-CT_ When ROI Included Two Volumes
VOI	Volume-of-Interest
VOI_SPECT_	VOI on SPECT matrix
VOI_SPECT-CT_	VOI on CT matrix
VOX_SPECT_	SPECT Voxels of V_SPECT_
VOX_SPECT-CT_	CT Voxels of V_SPECT-CT_
V_SPECT_	Volume Calculated from SPECT VOI
V_SPECT-CT_	Volume Calculated from CT VOI
WBS	Whole-Body Scintigraphy

## References

[1] Haugen B.R., Alexander E.K., Bible K.C., Doherty G.M., Mandel S.J., Nikiforov Y.E., Pacini F., Randolph G.W., Sawka A.M., Schlumberger M. (2016). 2015 American Thyroid Association Management Guidelines for Adult Patients with Thyroid Nodules and Differentiated Thyroid Cancer: The American Thyroid Association Guidelines Task Force on Thyroid Nodules and Differentiated Thyroid Cancer. Thyroid.

[2] Avram A.M., Giovanella L., Greenspan B., Lawson S.A., Luster M., Van Nostrand D., Peacock J.G., Ovcaricek P.P., Silberstein E., Tulchinsky M. (2022). SNMMI Procedure Standard/EANM Practice Guideline for Nuclear Medicine Evaluation and Therapy of Differentiated Thyroid Cancer: Abbreviated Version. J. Nucl. Med..

[3] Rault E., Vandenberghe S., Van Holen R., De Beenhouwer J., Staelens S., Lemahieu I. (2007). Comparison of Image Quality of Different Iodine Isotopes (I-123, I-124, and I-131). Cancer Biother. Radiopharm..

[4] Liu G., Li N., Li X., Chen S., Du B., Li Y. (2016). Thyroid Remnant Estimation by Diagnostic Dose (131)I Scintigraphy or (99m)TcO4(-) Scintigraphy after Thyroidectomy: A Comparison with Therapeutic Dose (131)I Imaging. BioMed Res. Int..

[5] Iwano S., Kato K., Nihashi T., Ito S., Tachi Y., Naganawa S. (2009). Comparisons of I-123 Diagnostic and I-131 Post-Treatment Scans for Detecting Residual Thyroid Tissue and Metastases of Differentiated Thyroid Cancer. Ann. Nucl. Med..

[6] Cooper D.S., Doherty G.M., Haugen B.R., Kloos R.T., Lee S.L., Mandel S.J., Mazzaferri E.L., McIver B., Pacini F., American Thyroid Association Guidelines Taskforce on Thyroid (2009). Revised American Thyroid Association management guidelines for patients with thyroid nodules and differentiated thyroid cancer. Thyroid.

[7] Pacini F., Schlumberger M., Dralle H., Elisei R., A Smit J.W., Wiersinga W., Taskforce T.E.T.C. (2006). European consensus for the management of patients with differentiated thyroid carcinoma of the follicular epithelium. Eur. J. Endocrinol..

[8] Carballo M., Quiros R.M. (2012). To Treat or Not to Treat: The Role of Adjuvant Radioiodine Therapy in Thyroid Cancer Patients. J. Oncol..

[9] Avram A.M., Fig L.M., Frey K.A., Gross M.D., Wong K.K. (2013). Preablation 131-I scans with SPECT/CT in postoperative thyroid cancer patients: What is the impact on staging?. J. Clin. Endocrinol. Metab..

[10] Durante C., Haddy N., Baudin E., Leboulleux S., Hartl D., Travagli J.P., Caillou B., Ricard M., Lumbroso J.D., De Vathaire F. (2006). Long-term outcome of 444 patients with distant metastases from papillary and follicular thyroid carcinoma: Benefits and limits of radioiodine therapy. J. Clin. Endocrinol. Metab..

[11] Schmidt D., Linke R., Uder M., Kuwert T. (2010). Five months’ follow-up of patients with and without iodine-positive lymph node metastases of thyroid carcinoma as disclosed by 131I-SPECT/CT at the first radioablation. Eur. J. Nucl. Med. Mol. Imaging.

[12] Rosenbaum-Krumme S.J., Wieduwilt M., Nagarajah J., Bockisch A., Jentzen W. (2012). Estimation of tumour mass in patients with differentiated thyroid carcinoma using serum thyroglobulin. Nuklearmedizin.

[13] Campennì A., Avram A.M., Verburg F.A., Iakovou I., Hänscheid H., de Keizer B., Petranović Ovčariček P., Giovanella L. (2023). The EANM guideline on radioiodine therapy of benign thyroid disease. Eur. J. Nucl. Med. Mol. Imaging.

[14] Schmidt M., Bartenstein P., Bucerius J., Dietlein M., Drzezga A., Herrmann K., Lapa C., Lorenz K., Musholt T.J., Nagarajah J. (2022). Individualized treatment of differentiated thyroid cancer: The value of surgery in combination with radioiodine imaging and therapy—A German position paper from Surgery and Nuclear Medicine. Nuklearmedizin.

[15] Lassmann M., Reiners C., Luster M. (2010). Dosimetry and thyroid cancer: The individual dosage of radioiodine. Endocr. Relat. Cancer.

[16] Mallick U., Harmer C., Yap B., Wadsley J., Clarke S., Moss L., Nicol A., Clark P.M., Farnell K., McCready R. (2012). Ablation with low-dose radioiodine and thyrotropin alfa in thyroid cancer. N. Engl. J. Med..

[17] Mortelmans L., Nuyts J., Van Pamel G., Van den Maegdenbergh V., De Roo M., Suetens P. (1986). A New Thresholding Method for Volume Determination by SPECT. Eur. J. Nucl. Med..

[18] Zaidi H. (1996). Comparative Methods for Quantifying Thyroid Volume Using Planar Imaging and SPECT. J. Nucl. Med..

[19] Lyra M., Striligas J., Gavrilelli M., Chatzijiannis C., Skouroliakou K. Thyroid Volume Determination by Single Photon Tomography and 3D Processing for Activity Dose Estimation. Proceedings of the IST IEEE Workshop on Imaging Systems and Techniques Proceedings.

[20] Pacilio M., Basile C., Shcherbinin S., Caselli F., Ventroni G., Aragno D., Mango L., Santini E. (2011). An innovative iterative thresholding algorithm for tumour segmentation and volumetric quantification on SPECT images: Monte Carlo-based methodology and validation. Med. Phys..

[21] Pierre F., Amendola M., Bigeard C., Ruel T., Villard P.-F. (2021). Segmentation with Active Contours. Image Process Line.

[22] Al-Ameen Z., Sulong G., Rehman A., Al-Dhelaan A., Al-Rodhaan M. (2020). A Review of Image Segmentation Using MATLAB Environment. IEEE Access.

[23] Verma S., Khare D., Gupta R., Chandel G.S. (2012). Analysis of Image Segmentation Algorithms Using MATLAB. Proceedings of the Third International Conference on Trends in Information, Telecommunication and Computing.

[24] Rogowska J. (2009). Overview and Fundamentals of Medical Image Segmentation. Handbook of Medical Image Processing and Analysis.

[25] Otsu N. (1979). A threshold selection method from gray-level histogram. IEEE Trans. Syst. Man Cybern..

[26] Soret M., Bacharach S.L., Buvat I. (2007). Partial-Volume Effect in PET Tumor Imaging. J. Nucl. Med..

[27] Marquis H., Willowson K.P., Bailey D.L. (2023). Partial Volume Effect in SPECT & PET Imaging and Impact on Radionuclide Dosimetry Estimates. Asia Ocean. J. Nucl. Med. Biol..

[28] Michael K., Hadjiconstanti A., Lontos A., Demosthenous G., Frangos S., Parpottas Y. (2023). A Neck-Thyroid Phantom with Small Sizes of Thyroid Remnants for Postsurgical I-123 and I-131 SPECT/CT Imaging. Life.

[29] Boening G., Pretorius P.H., King M.A. (2006). Study of Relative Quantification of Tc-99m with Partial Volume Effect and Spillover Correction for SPECT Oncology Imaging. IEEE Trans. Nucl. Sci..

[30] Matsubara K., Ibaraki M., Shimada H., Ikoma Y., Suhara T., Kinoshita T., Ito H. (2016). Impact of Spillover from White Matter by Partial Volume Effect on Quantification of Amyloid Deposition with [11C]PiB PET. NeuroImage.

[31] MATLAB (2022). MATLAB Release 2022a.

[32] Michael K., Frangos S., Iakovou I., Lontos A., Demosthenous G., Parpottas Y. (2024). The Impact of Dual and Triple Energy Window Scatter Correction on I-123 Postsurgical Thyroid SPECT/CT Imaging Using a Phantom with Small Sizes of Thyroid Remnants. Life.

[33] Hadjiconstanti A., Michael K., Frangos S., Demosthenous G., Lyra M. The Impact of Two Scatter Correction Methods on I-131 AC-SPECT Images Using an Anthropomorphic Phantom with Variable Sizes of Thyroid Remnants. Proceedings of the 2020 7th International Conference on Biomedical and Bioinformatics Engineering.

[34] Lagerburg V., de Nijs R., Holm S., Svarer C. (2012). A Comparison of Different Energy Window Subtraction Methods to Correct for Scatter and Downscatter in I-123 SPECT Imaging. Nucl. Med. Commun..

[35] Mandel S.J., Shankar L.K., Benard F., Yamamoto A., Alavi A. (2001). Superiority of Iodine-123 Compared with Iodine-131 Scanning for Thyroid Remnants in Patients with Differentiated Thyroid Cancer. Clin. Nucl. Med..

[36] Siddiqi A., Foley R.R., Britton K.E., Sibtain A., Plowman P.N., Grossman A.B., Monson J.P., Besser G.M. (2001). The Role of 123I-Diagnostic Imaging in the Follow-up of Patients with Differentiated Thyroid Carcinoma as Compared to 131I-Scanning: Avoidance of Negative Therapeutic Uptake due to Stunning. Clin. Endocrinol..

[37] Hilditch T.E., Dempsey M.F., Bolster A.A., McMenemin R.M., Reed N.S. (2002). Self-Stunning in Thyroid Ablation: Evidence from Comparative Studies of Diagnostic 131I and 123I. Eur. J. Nucl. Med. Mol. Imaging.

[38] Benfante V., Stefano A., Ali M., Laudicella R., Arancio W., Cucchiara A., Caruso F., Cammarata F.P., Coronnello C., Russo G. (2023). An Overview of In Vitro Assays of 64Cu-, 68Ga-, 125I-, and 99mTc-Labelled Radiopharmaceuticals Using Radiometric Counters in the Era of Radiotheranostics. Diagnostics.

[39] Ali M., Benfante V., Di Raimondo D., Laudicella R., Tuttolomondo A., Comelli A. (2024). A Review of Advances in Molecular Imaging of Rheumatoid Arthritis: From In Vitro to Clinic Applications Using Radiolabeled Targeting Vectors with Technetium-99m. Life.

[40] Alongi P., Stefano A., Comelli A., Spataro A., Formica G., Laudicella R., Lanzafame H., Panasiti F., Longo C., Midiri F. (2022). Artificial Intelligence Applications on Restaging [18F]FDG PET/CT in Metastatic Colorectal Cancer: A Preliminary Report of Morpho-Functional Radiomics Classification for Prediction of Disease Outcome. Appl. Sci..

